# Severe sepsis caused by *Aeromonas hydrophila *in a patient using tocilizumab: a case report

**DOI:** 10.1186/1752-1947-5-499

**Published:** 2011-10-05

**Authors:** Kenji Okumura, Fumihiro Shoji, Masaki Yoshida, Atsushi Mizuta, Ichiro Makino, Hidefumi Higashi

**Affiliations:** 1 Department of Surgery, Nippon Steel Yawata Memorial Hospital, 1-1-1, Harunomachi, Yahatahigashi-ku, Kitakyushu 805-8508, Japan

## Abstract

**Introduction:**

*Aeromonas *species do not commonly cause disease in humans. However, when disease is seen, it often occurs in patients with underlying immunosuppression or malignancy and has a high fatality rate.

**Case presentation:**

A 72-year-old Japanese woman with rheumatoid arthritis treated with tocilizumab (which has an immunosuppressive effect) presented with severe epigastric pain. She had a fever with chills, hypotension and jaundice. She was diagnosed with acute suppurative cholangitis and treated with cefoperazone-sulbactam and an endoscopic drainage was performed. Jaundice was slightly improved, but the shock state and inflammatory reactions were prolonged as typical of septic shock. On the second day after admission, an electrocardiogram showed ST segment elevation and echocardiography showed ventricular wall dysfunction. Coronary arteries were patent in coronary angiography and she was diagnosed with stress-induced cardiomyopathy. Blood cultures showed *Aeromonas hydrophila*. A stool culture was negative for *A. hydrophila*. On day six, her white blood cell count and neutrophils were normalized and cefoperazone-sulbactam treatment was halted. Left ventricular function normalized on day twelve and a laparoscopic cholecystectomy for cholelithiasis was performed on the 16th day of hospitalization. A culture from the bile showed *A. hydrophila*. Eighteen days after surgery, tocilizumab treatment was restarted and there were no complications. Two months after restarting tocilizumab, our patient is stable without any serious events.

**Conclusion:**

We present a rare case of *A. hydrophila *sepsis and acute suppurative cholangitis in an elderly patient with gallstones and rheumatoid arthritis using tocilizumab. This clinical course may suggest that preemptive treatment for cholelithiasis prior to using molecular-targeting agents might be feasible in elderly patients.

## Introduction

*Aeromonas hydrophila *is distributed widely in fresh and salt water, and is also found in food, treated drinking water, domestic water supplies and hospital water supply systems [1,2]. Typically, patients acquire *Aeromonas *species by oral consumption or direct contact with contaminated water or seafood. Thus, gastroenteritis and mild-to-moderate soft-tissue infections are the most common presentations. In immunocompromised individuals, such as patients with cirrhosis, malignant diseases, chronic renal failure, diabetes mellitus or steroid use, Aeromonas spp. cause substantial mortality from a wide spectrum of infections. These include hepatobiliary infection, invasive skin and soft-tissue infections, primary bacteremia, burn infections, pleuropulmonary infection, meningitis and endocarditis [1,2]. The species *A. hydrophila*, *A. caviae*, and *A. veronii biovar sobria *account for more than 85% of human infections [1,2]. *Aeromonas *infection is often polymicrobial and fatality rates range from 28% to 46% in cases of bacteremia, mostly caused by *A. hydrophila *and *A. veronii biovar sobria *[1-3]. Tocilizumab, developed as a treatment of rheumatoid arthritis, is a humanized anti-interleukin-6 receptor monoclonal antibody, and can cause infections as adverse events. We report here a rare case of *A. hydrophila *sepsis and acute suppurative cholangitis in an elderly patient with rheumatoid arthritis using tocilizumab.

## Case presentation

**Figure 1:**
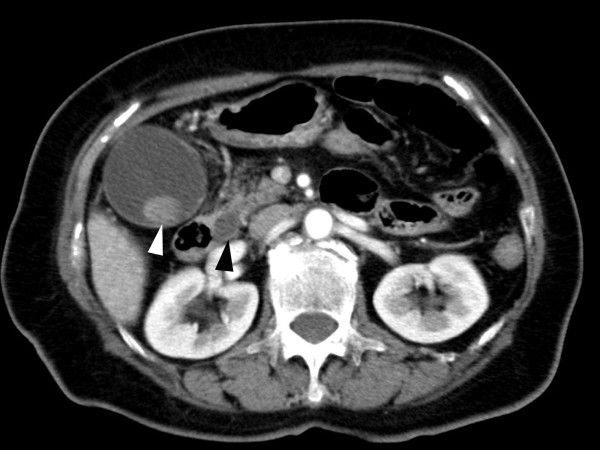
**CT scan showing cholelithiasis (white arrow), choledocholithiasis and dilated common bile duct (black arrow) with a calcified stone**.

A 72-year-old Japanese woman was admitted with severe epigastric pain and vomiting. She had a history of rheumatoid arthritis treated with tocilizumab every four weeks. On examination, she had a temperature of 39.2°C with chills. Her blood pressure was 77/46 mmHg, with a heart rate of 96 bpm. She was jaundiced, but had no signs of palmar erythema, ankle edema, finger clubbing, spider nevi or evidence of skin injury or infection. She had abdominal pain with right upper-quadrant discomfort and a positive Murphy's sign. Laboratory results showed the following: white blood cell count 7600/μL (normal range: 3100-8800 μL), neutrophils 97.7% (normal range: 50-70%), C-reactive protein 2.16 mg/dL (normal range: 0-0.25 mg/dL), a platelet count of 150 × 10^9^/μL (normal range: 140-440 × 10^9^/μL), aspartate transaminase 266 IU/L (normal range: 13-33 IU/L), alanine transaminase 432 IU/L (normal range: 6-27 IU/L), and alkaline phosphatase 890 IU/L (normal range: 115-359 IU/L). In addition, her total bilirubin was 7.0 mg/dL (normal range: 0.3-1.5 mg/dL), direct bilirubin 5.2 mg/dL (normal range: 0-0.2 mg/dL), gamma glutamyl transferase, 342 IU/L (normal range: 10-60 IU/L), protein 59 g/L (normal range: 67-83 g/L) and albumin 33 g/L (normal range: 40-50 g/L). An abdominal ultrasonography revealed an enlarged gall bladder with stones, and dilation of her intrahepatic and common bile ducts. Computed tomography (CT) showed cholelithiasis, choledocholithiasis, dilated common bile duct with a calcified stone and normal liver shape (Figure 1). Our patient was diagnosed with sepsis due to acute suppurative cholangitis. Blood samples were collected immediately and cefoperazone-sulbactam (1 g intravenously every 12 hours) was started for biliary tract infection. Endoscopic retrograde cholangiopancreatography was performed and two stones were drained along with sludge. Vasopressors were used to manage shock. Jaundice was slightly improved, but the shock state was prolonged as is typical of this condition. On the second day following admission, she vomited and exhibited hypotension with bradycardia. An electrocardiogram showed ST segment elevation at I and aVL. Echocardiography showed left posterolateral ventricular wall dysfunction. Coronary angiography showed patent coronary arteries and she was diagnosed with stress-induced cardiomyopathy. Blood cultures were positive for *A. hydrophila *and *Klebsiella pneumoniae*, both of which were susceptible to cefoperazone-sulbactam. *A. hydrophila *was resistant to penicillin, ampicillin, ampicillin-sulbactam, and first- and second-generation cephalosporins, and susceptible to piperacillin, third-generation cephalosporins, aminoglycosides, carbapenems, tetracyclines, trimethoprim-sulfamethoxazole and fluoroquinolones. A stool culture was negative for *A. hydrophila *and no malignancy, cirrhosis, chronic renal failure or diabetes mellitus was evident in additional investigations. On day six, her white blood cell count and the percentage of neutrophils were normalized and cefoperazone-sulbactam treatment was halted. Left ventricular function normalized on day twelve and laparoscopic cholecystectomy for cholelithiasis was performed on the 16th day of hospitalization. A culture from her bile showed only *A. hydrophila*. Eighteen days after surgery, tocilizumab treatment was restarted and there were no complications. Two months after restarting tocilizumab, our patient is stable without any serious events.

## Discussion

*Aeromonas *spp. are ubiquitous mobile Gram-negative rods found in water sources. They cause a wide range of human illness; possible routes of transmission include contaminated food and exposure of wounds to environments that contain the pathogen [1,4]. Severe *A. hydrophila *infections usually involve immunocompromised people with chronic illness [1,2]. *Aeromonas spp. *produce a beta-lactamase, which makes them resistant to ampicillin and first-generation cephalosporins. The antimicrobial agents most active against *Aeromonas *are the third-generation cephalosporins, imipenem and fluoroquinolones [5,6].

The frequency of acute suppurative cholangitis due to *Aeromonas *is low (less than 3%) [1,3]. *Aeromonas *hepatobiliary infections are commonly associated with cholelithiasis, choledocholithiasis, malignancy, other immunocompromised conditions and recent surgical procedures [1-3].

Tocilizumab, used for the treatment of rheumatoid arthritis, is a humanized monoclonal antibody against interleukin-6, a cytokine that plays a multifunctional and important role in the immune response [7]. Infection was the most common adverse event associated with tocilizumab in clinical trials [7,8]. Serious bacterial, viral or fungal infections can occur when using tocilizumab, such as tuberculosis [8]. The rate of serious infections was 3.6 events per 100 patient-years, but the overall rate of fatal infections was low (0.13 events per 100 patient-years) [7].

In this immunocompromised patient receiving treatment with tocilizumab and with known cholelithiasis, sepsis with *A. hydrophila *and *Klebsiella pneumoniae *developed secondary to pyogenic cholangitis due to choledocholothiasis. No gastrointestinal symptoms preceded or were concurrent with sepsis, and a stool culture was negative for *A. hydrophila*. There were no signs of soft tissue infection and no previous episodes of treating infections with antibiotics during the past year. Only *A. hydrophila *was detected in the gall bladder after the cholangitis had improved. These results may suggest that *A. hydrophila *was carried in the biliary tract and that stone obstruction of the biliary tract caused sepsis with ascending infection of *Klebsiella pneumoniae*. No other infectious pathway seems likely.

In healthy individuals, bacteria are not found in the gall bladder, but in patients with gallstones the percentage of positive cultures depends upon the severity of the disease and age [9]. Thus, preemptive treatment for cholelithiasis prior to using molecular-targeting agents might be feasible in elderly patients.

## Conclusion

We present a rare case of *A. hydrophila *sepsis and acute suppurative cholangitis in a patient with gallstones and rheumatoid arthritis using tocilizumab.

## Consent

Written informed consent was obtained from the patient for publication of this manuscript and the accompanying image. A copy of the written consent is available for review by the Editor-in-Chief of this journal.

## Competing interests

The authors declare that they have no competing interests.

## Authors' contributions

KO undertook the gathering of information for this case and was a major contributor in writing the manuscript. FS conceived the manuscript and was a major contributor to the manuscript. All authors read and approved the final manuscript.
